# Laboratory Predictors of COVID-19 Mortality: A Retrospective Analysis from Tongji Hospital in Wuhan

**DOI:** 10.1155/2021/6687412

**Published:** 2021-02-23

**Authors:** Ting Zheng, Xinxin Liu, Yingying Wei, Xinlu Li, Bing Zheng, Quan Gong, Lingli Dong, Jixin Zhong

**Affiliations:** ^1^Department of Immunology, School of Medicine, Yangtze University, Jingzhou, Hubei 434023, China; ^2^Department of Rheumatology and Immunology, Tongji Hospital, Tongji Medical College, Huazhong University of Science and Technology, Wuhan, Hubei 430030, China

## Abstract

**Background:**

Novel coronavirus disease 2019 (COVID-19), an acute respiratory disease caused by severe acute respiratory syndrome coronavirus 2 (SARS-CoV-2), rapidly progressed to a global pandemic. Currently, there are limited effective medications approved for this deadly disease.

**Objective:**

To investigate the potential predictors of COVID-19 mortality and risk factors for hyperinflammation in COVID-19.

**Methods:**

Retrospective analysis was carried out in 1,149 patients diagnosed with COVID-19 in Tongji Hospital, Wuhan, China, from 1/13/2020 to 3/15/2020.

**Results:**

We found significant differences in the rates of hyperuricemia (OR: 3.17, 95% CI: 2.13-4.70; *p* < 0.001) and hypoalbuminemia (OR: 5.68, 95% CI: 3.97-8.32; *p* < 0.001) between deceased and recovered patients. The percentages of hyperuricemia in deceased patients and recovered patients were 23.6% and 8.9%, respectively, which were higher than the reported age-standardized prevalence of 6.2% in Chinese population. Of note, the percentages of both IL-6 and uric acid levels in survived COVID-19 patients were above 90%, suggesting that they might be good specificity for indicators of mortality in COVID-19 patients. The serum level of uric acid (UA) was positively associated with ferritin, TNF-*α*, and IL-6 but not with anti-inflammatory cytokine IL-10. In addition, the levels of these proinflammatory cytokines in COVID-19 patients showed a trend of reduction after uric acid lowering therapy.

**Conclusions:**

Our results suggest that uric acid, the end product of purine metabolism, was increased in deceased patients with COVID-19. In addition, the serum level of uric acid was positively associated with inflammatory markers. Uric acid lowering therapy in COVID-19 patients with hyperuricemia may be beneficial.

## 1. Introduction

Coronavirus disease 19 (COVID-19) caused by the ribonucleic acid (RNA) virus severe acute respiratory syndrome coronavirus 2 (SARS-CoV-2), emerged in Wuhan City, Hubei Province, China, has spread rapidly across the world. At the time of drafting this manuscript (Oct. 19, 2020), the worldwide death toll from the COVID-19 pneumonia eclipsed 1,000,000 [1], and the number of people infected continues to slowly climbed upward. Although predictors such as high-sensitivity C-reactive protein (hsCRP), aspartate aminotransferase (AST), and D-dimer for mortality of COVID-19 patients had been determined [2], more risk predictors and prognostic factors still desperately needed to been found to improve the treatment programs for infected patients, especially for patients with other underlying diseases (such as identified risk factors indicator cardiac troponin I to preexisting concurrent cardiovascular or cerebrovascular diseases [3]; BMI for COVID-19 severity in the population living with diabetes in hospital admission [4]).

## 2. Materials and Methods

### 2.1. Participants and Clinical Variables

We analyzed and observed serological tests result a number of laboratory parameters may serve as predictors of disease progression in 1,149 in patients diagnosed with COVID-19 in Tongji Hospital, Wuhan, China, from 1/13/2020 to 3/15/2020 and performed statistical analysis to estimate ORs and 95% CIs of mortality. The correlation between serum uric acid and other inflammatory factors and the content of these inflammatory factors before and after treatment were compared.

### 2.2. Statistical Analysis

Categorical variables were reported as percentages. Combined odds ratios (ORs) and 95% CIs were evaluated as effect size using uni- and multivariate analyses. We used linear regression to evaluate any association between two variables. *p* values less than 0.05 were considered statistically significant.

### 2.3. Ethics

The study was performed in accordance with the ethical standards laid down in the Declaration of Helsinki. Our work has been reviewed and approved by Tongji Hospital Ethics Committee.

## 3. Results

In univariate analyses, patients who died from COVID-19 had higher hyperinflammation markers than patients who survived: lactate dehydrogenase (LDH, OR: 25.14, 95% CI: 17.06-37.53; *p* < 0.0001), AST (OR: 5.08, 95% CI: 3.67-7.05; <0.0001), alanine aminotransferase (ALT, OR: 1.52, 95% CI: 1.07-2.14; *p* = 0.018), ferritin (OR: 12.92, 95% CI: 5.74-37.0; *p* < 0.0001), and inflammatory cytokines TNF-*α* (OR: 4.34, 95% CI: 2.90-6.59; *p* < 0.0001), Interleukin-6 (IL-6, OR: 68.63, 95% CI: 31.02-182.30; *p* < 0.0001), Interleukin-8 (IL-8, OR: 7.60, 95% CI: 4.26-13.80; *p* < 0.0001), and anti-inflammatory cytokines Interleukin-10 (IL-10, OR: 8.06, 95% CI: 3.56-20.75; *p* < 0.0001). In addition to these previously confirmed indicators of COVID-19 disease severity [5, 6], we found significant differences in the rates of hyperuricemia (OR: 3.17, 95% CI: 2.13-4.70; *p* < 0.001) and hypoalbuminemia (OR: 5.68, 95% CI: 3.97-8.32; *p* < 0.001) between deceased and recovered patients. The percentages of hyperuricemia in deceased patients and recovered patients were 23.6% and 8.9%, respectively, which are higher than the reported age-standardized prevalence of 6.2% in Chinese population [7]. Of note, the percentages of both IL-6 and uric acid (UA) levels in survived COVID-19 patients were above 90%, suggesting that these two factors have a good specificity when used as indicators for COVID-19 mortality.

**Figure 1:**
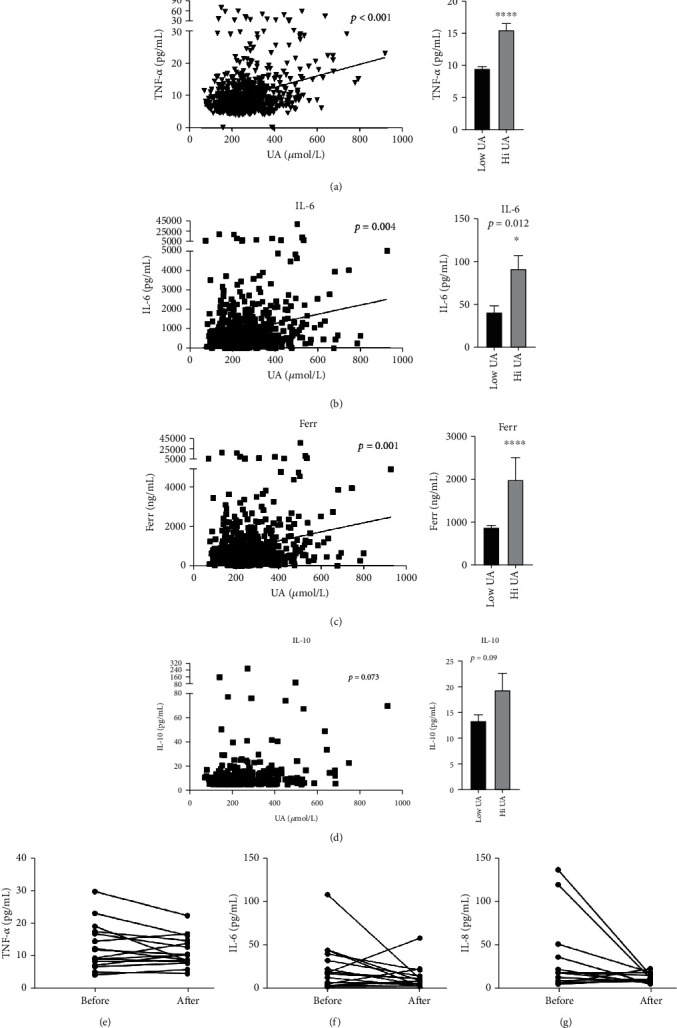
Uric acid is associated with serum levels of TNF-*α*, IL-6, and ferritin in COVID-19 patients. (a) Left, serum uric acid (UA) is associated with serum TNF-*α* level; right, serum TNF-*α* level in patients with a UA level of lower or higher than 400 *μ*mol/L. (b) Left, association between serum UA and IL-6 levels; right, serum IL-6 level in patients with a UA level of lower or higher than 400 *μ*mol/L. (c) Left, association between serum UA and ferritin levels; right, serum ferritin level in patients with a UA level of lower or higher than 400 *μ*mol/L. (d) Left, association between serum UA and IL-10 levels; right, serum IL-10 level in patients with a UA level of lower or higher than 400 *μ*mol/L. (e) Serum TNF-*α* level before and after uric acid lowering therapy. (f) Serum IL-6 level before and after uric acid lowering therapy. (g) Serum IL-8 level before and after uric acid lowering therapy.

Interestingly, the serum level of uric acid is positively associated with ferritin, TNF-*α*, and IL-6 but not with anti-inflammatory cytokine IL-10 (Figures 1(a)–1(d)). Patients with a serum uric acid level over 400 *μ*mol/L had higher serum levels of TNF-*α*, IL-6, and ferritin. To investigate if the increase of uric acid contributes to the hyperinflammatory status in COVID-19, we analyzed cytokine levels before and after administration of uric acid lowering agents (febuxostat or benzbromarone). A total of 16 COVID-19 patients with an acute gout attack during the hospital stay were examined for cytokine profile before and after the treatment with uric acid lowering therapy. After an average of 7 days of therapy, there was a trend toward reduced serum levels of IL-6, IL-8, and TNF-*α* (Figures 1(e)–1(g)). There were 9 out of the 16 patients taking uric acid lowering agents that had a serum uric acid level over 400 *μ*mol/L. While 8 out of these 9 patients recovered from COVID-19, only 74 out of the 121 hyperuricemia patients without uric acid lowering agents survived (88.9% vs. 61.2%; *p* = 0.096).

## 4. Discussion

We observed in this study that deceased COVID-19 patients had a higher rate of hyperuricemia. Given the fact that biomarkers of tissue damage such as LDH, AST, ALT, and ferritin were increased in severely ill or deceased COVID-19 patients (Table 1 and references [5, 6]), increased percentages of hyperuricemia in COVID-19 patients, especially in deceased patients, may be a result of tissue damage/cell death. Uric acid has been characterized as a danger signal to alarm immune system to cell injury and initiate immune responses to clear damaged cells/tissues [8]. Uric acid is a degradation product of purines and presents in the blood at a high concentration approaching to the saturation threshold. In the event of tissue damage, intracellular stores of uric acid are released out of the cells, which may cause hyperuricemia and form crystals. Crystallized form of uric acid is a strong inducer of NLRP3 inflammasome, inciting robust inflammation. Nucleated uric acid is able to stimulate the production of inflammatory cytokines such as IL-1, TNF-*α*, and IL-6 in gout, a classic disease caused by hyperuricemia. In this case series, we observed positive correlations between uric acid and inflammatory markers in patients with COVID-19. In addition, uric acid lowering treatment with febuxostat and/or benzbromarone reduced levels of proinflammatory cytokines in COVID-19 patients. There was also a trend toward reduced mortality rate in hyperuricemia patients with uric acid lowering therapy. These results suggest that uric acid lowering therapy may be beneficial in COVID-19 patients with hyperuricemia. However, a further investigation with larger sample size and randomized controls is required to confirm the beneficial effects of uric acid lowering therapy in reducing proinflammatory cytokines.

In summary, uric acid released from the injured tissues/cells may serve as a danger signal to amplify the hyperinflammatory response in severe COVID-19 cases. Uric acid lowering therapy in COVID-19 patients with hyperuricemia patients may be able beneficial.

## Figures and Tables

**Table 1:** Laboratory parameters in 1,149 COVID-19 patients hospitalized in Tongji hospital between 1/13/2020 and 3/15/2020.

	Deceased, *n* (%)	Survived, *n* (%)	OR (95% CI)	*p*
Age (years)				
<60, *n* (%)	41 (19.0)	522 (55.9)	1.00	—
≥60, *n* (%)	175 (81.0)	411 (44.1)	3.36 (2.36-4.89)	<0.0001
Gender				
Male, *n* (%)	145 (67.1)	477 (47.9)	2.23 (1.63-3.09)	<0.0001
Female, *n* (%)	71 (32.9)	486 (52.1)	1.00	—
IL-6 (pg/mL)				
>150, *n* (%)	61 (43.3)	6 (1.1)	68.63 (31.02-182.30)	<0.0001
≤150, *n* (%)	80 (56.7)	540 (98.9)	1.00	—
IL-10 (pg/mL)				
> 15, *n* (%)	72 (68.6)	44 (29.7)	8.06 (3.56-20.75)	<0.0001
≤ 15, *n* (%)	33 (31.4)	104 (70.3)	1.00	—
IL-8 (pg/mL)				
> 100, *n* (%)	32 (24.8)	22 (4.2)	7.60 (4.26-13.80)	<0.0001
≤ 100, *n* (%)	97 (75.2)	507 (95.8)	1.00	—
TNF-*α* (pg/mL)				
> 100, *n* (%)	83 (65.4)	159 (30.3)	4.34 (2.90-6.59)	<0.0001
≤ 100, *n* (%)	44 (34.6)	366 (69.7)	1.00	—
Uric acid (*μ*mol/L)				
> 400, *n* (%)	48 (23.6)	82 (8.9)	3.17 (2.13-4.70)	<0.0001
≤ 400, *n* (%)	155 (76.4)	840 (91.1)	1.00	—
Ferritin (ng/mL)				
> 400 (M)/300 (F), *n* (%)	123 (96.1)	337 (65.6)	12.92 (5.74-37.0)	<0.0001
≤ 400 (M)/300 (F), *n* (%)	5 (3.9)	177 (34.4)	1.00	—
Albumin (g/L)				
≥ 35, *n* (%)	40 (19.9)	537 (58.4)	1.00	—
< 35, *n* (%)	161 (80.1)	382 (41.6)	5.68 (3.97-8.32)	<0.0001
LDH (U/L)				
> 450, *n* (%)	123 (62.4)	57 (6.2)	25.14 (17.06-37.53)	<0.0001
≤ 450, *n* (%)	74 (34.6)	862 (93.8)	1.00	—
ALT (U/L)				
> 40, *n* (%)	57 (28.4)	189 (20.6)	1.52 (1.07-2.14)	0.018
≤ 40, *n* (%)	144 (71.6)	728 (79.4)	1.00	—
AST (U/L)				
> 40, *n* (%)	102 (50.7)	153 (16.7)	5.08 (3.67-7.05)	<0.0001
≤ 40, *n* (%)	99 (49.3)	764 (83.3)	1.00	—
AST/ALT				
> 1, *n* (%)	159 (79.1)	533 (60.3)	2.48 (1.74-3.61)	<0.0001
≤ 1, *n* (%)	42 (20.9)	364 (39.7)	1.00	—

ALT: alanine aminotransferase; AST: aspartate aminotransferase; IL-6: interleukin 6; IL-8: interleukin 8; IL-10: interleukin 10; LDH: lactate dehydrogenase; TNF-*α*: tumor necrosis factor *α*.

## Data Availability

Derived data supporting the findings of this study are available from the corresponding author (JZ, LD, QG) on request.
